# Genetic liability to obesity and peptic ulcer disease: a Mendelian randomization study

**DOI:** 10.1186/s12920-022-01366-x

**Published:** 2022-10-04

**Authors:** Zhoubin Li, Heng Chen, Ting Chen

**Affiliations:** 1 Department of Lung Transplantation and General Thoracic Surgery, The First Affiliated Hospital, Zhejiang University School of Medicine, Hangzhou, China Zhejiang Province; 2 Department of Cardiology, The First Affiliated Hospital, Zhejiang University School of Medicine, 79 Qingchun Road, Hangzhou, 310003 Zhejiang Province China; 3 Alibaba-Zhejiang University Joint Research Center of Future Digital Healthcare, Hangzhou, China

**Keywords:** Mendelian randomization, Causal association, Body mass index, Waist-to-hip ratio, Peptic ulcer disease

## Abstract

**Background:**

Epidemiological evidence relating obesity to peptic ulcer disease (PUD) has been mixed. Here we sought to determine the causality in the association of obesity with PUD risk using the Mendelian randomization (MR) approach.

**Methods:**

This study was based on summary-level data for body mass index (BMI), waist-to-hip ratio (WHR), and PUD derived from large genome-wide association studies (GWASs). Single nucleotide polymorphisms significantly associated with BMI and WHR (*P* < 5 × 10^–8^) were leveraged as instrumental variables. Causal estimates were pooled using several meta-analysis methods. In addition, multivariable MR was employed to account for covariation between BMI and WHR, as well as to explore potential mediators.

**Results:**

Genetically predicted higher BMI has a causal effect on PUD, with an OR of 1.34 per SD increase in BMI (~ 4.8 kg/m^2^) (*P* = 9.72 × 10^–16^). Likewise, there was a 35% higher risk of PUD (*P* = 2.35 × 10^–10^) for each SD increase in WHR (0.09 ratio). Complementary analyses returned consistent results. Multivariable MR demonstrated that adjustment for WHR largely attenuated the BMI-PUD association. However, the causal association of WHR with PUD risk survived adjustment for BMI. Both the associations remained robust upon adjustment for several traditional risk factors. Replication analyses using different instrumental variants further strengthened the causal inference. Besides, we found no evidence for the causal association in the reverse analyses from PUD to BMI/WHR.

**Conclusions:**

This MR study revealed that obesity (notably abdominal obesity) is causally associated with higher PUD risk. Programs aimed at weight loss may represent therapeutic opportunities for PUD.

**Supplementary Information:**

The online version contains supplementary material available at 10.1186/s12920-022-01366-x.

## Introduction

Peptic ulcer disease (PUD) is a common gastrointestinal disorder that significantly impacts the quality of life [1, 2]. Management has become more challenging than ever before due to the increasing prevalence of PUD not caused by the use of NSAIDs or *Helicobacter Pylori* infection [2]. The identification of modifiable risk factors for PUD is clinically important in reducing the burden of the disease.

Obesity has been associated with an alteration in gut microbiota [3], gut inflammation, and the breakdown of the gastrointestinal mucosal epithelial barrier [4]. Epidemiological studies suggested that obesity may increase the incidence of PUD [5–7]. However, evidence of the association was contradictory. In another observational study, Jeung Hui Pyo et al*.* reported that obesity was not related to PUD [8] upon multiple adjustment. In addition, observational associations may be biased by reverse causation and residual confounders, thus distorting true relationships.

Clarifying the causal link between obesity and PUD may offer new avenues for the treatment against PUD. Mendelian randomization (MR) has been widely used as an epidemiological tool for causality inference in associations of exposures with outcomes [9]. With genetic variants leveraged as instruments, this technique can minimize the biases inherent in observational studies, thereby strengthening the causal inference. The present study sought to evaluate the potential causal effect of total obesity and abdominal obesity on PUD risk by the MR approach.

## Methods

### Study design

First, we performed two-sample MR analyses to assess the effect of body mass index (BMI) (total obesity) and waist-to-hip ratio (WHR) (abdominal obesity) as exposures on PUD as an outcome trait. Then, we performed reverse MR analyses, where PUD was used as exposure and obesity characteristics (BMI/WHR) were used as outcome. The following three key assumptions should be considered when performing MR analysis. First, instrumental variables (IVs) are linked to BMI or WHR at a genome-wide significance level; Second, IVs are not associated with potential confounding factors; Third, IVs should lead to PUD exclusively via the selected exposure. The current study did not require specific ethical approval or written informed consent.

### Data sources and instruments variables (IVs) selection

Summary-level data for PUD was derived from a genome-wide association study (GWAS) including up to 16,666 cases and 439,661 controls from UK biobank (Table 1) [10]. Individuals with gastric ulcer, duodenal ulcer, other site peptic ulcer, or astro-jejunal were defined as PUD cases [10].

**Table 1:** Detailed information on data sources

Phenotype	Study or consortium	Ancestry	Sample size	Cases	Adjustment
PUD	UKBiobank	European	456,327	16,666	Sex, age and 20 ancestry principal components
BMI	UKBiobank + GIANT	European	806,834	–	Age, age-squared, sex and principal components 1–5
WHR	UKBiobank + GIANT	European	697,734	–	Age, age-squared, sex and principal components 1–5
H.P. infection	UKBiobank	European	462,933	1,329	Age, sex, 10 genetic principal components, and genotyping batch
T2D	DIAGRAM	European	149,821	34,840	Study-specific covariates, including indicators of population structure
LDL-C, HDL-C, TC, TG	GLGC	European	188,578	–	Age, age^2^, and sex
Smoking, alcohol use	GSCAN	European	1,232,091	–	Age, sex, age × sex interaction, and the first ten genetic principle components
BMI	GIANT	European	339,224	–	Age, age squared, and any necessary study-specific covariates (e.g. genotype-derived principal components)
WHR	GIANT	European	224,459	–	Age, age^2^, and other study-specific covariates

*PUD* peptic ulcer disease; *BMI* body mass index; *WHR* waist-to-hip ratio; *H.P.* Helicobacter Pylori; *T2D* type 2 diabetes mellitus; *LDL-C* low-density lipoprotein cholesterol; *HDL-C* high-density lipoprotein cholesterol; *TC* total cholesterol; *TG* triglyceride; *GIANT* Genetic Investigation of ANthropometric Traits; *DIAGRAM* DIAbetes Genetics Replication and Meta-analysis; *GLGC* global lipids genetics consortium; *GSCAN* GWAS & Sequencing Consortium of Alcohol and Nicotine use

Primary analyses were performed using genome-wide significant (*P* < 5 × 10^–8^) single nucleotide polymorphisms (SNPs) identified from the largest GWAS that combined data from UK biobank and the Genetic Investigation of Anthropometric Traits (GIANT) consortium, including up to 694,649 individuals of European descent (Table 1) [11]. We set a cut-off for minor allele frequency to > 1%, leaving 85,044 and 39,427 SNPs for BMI and WHR (unadjusted for BMI), respectively. To obtain valid IVs, these SNPs were then pruned at r^2^ < 0.001 across a window size of 10000 kb (based on the 1000 Genomes Project population [12]) to avoid bias of linkage disequilibrium. For those SNPs not available in the outcome dataset, proxies (r^2^ > 0.8) were found by searching the publicly available website (http://snipa.helmholtz-muenchen.de/snipa3/) (Additional file 1: Table S1). Finally, 531 and 343 SNPs were included for BMI and WHR, respectively (Additional file 1: Table S2 and 3). In addition, we calculated F-statistics to assess whether there was a weak IV bias ($F={R}^{2}\frac{N-2}{1-{R}^{2}}$). R^2^ refers to the percentage of the variation explained by SNPs and is calculated as described by Shim et al. [13]; N represents the total sample size [14].

However, there was a sample overlap (UK Biobank mainly) in the participants included in the primary analyses (Table 1). To test the robustness of the results, we then retrieved summary-level data for BMI and WHR from other GWASs where only individuals of the GIANT consortium were included (339,224 samples for BMI and 224,459 samples for WHR), so that there would be no sample overlap with the outcome data (Table 1) [15, 16]. These SNPs underwent similar quality-control steps, leaving 78 SNPs and 29 SNPs for BMI and WHR, respectively. Considering 2 SNPs for BMI not available in the outcome datasets with no suitable proxies found, both SNPs were excluded from the study. Here, we provided the characteristics of the remaining 76 SNPs and 29 SNPs in Additional file 1: Table S4 and S5, with R^2^ and F-statistics calculated as well.

In the reverse analyses, we extracted 8 independent SNPs as IVs for PUD using the above method. SNPs not found in the outcome datasets were replaced with their proxies if any (Additional file 1: Table S6). Detailed information for these SNPs were provided in Additional file 1: Table S7.

### Mendelian randomization analyses

Inverse-variance weighted (IVW) in the multiplicative random-effects model was employed as the main method given the presence of heterogeneity among SNPs [17, 18]. A series of complementary analyses were conducted: The weighted median [19] and MR-Egger [20] provided more conservative causal estimates; and the MR-pleiotropy residual sum and outlier (MR-PRESSO) methods [21] was employed to identify pleiotropic outliers that may bias the results. Heterogeneity among IVs was measured by the heterogeneity Q test and I^2^ statistics. Horizontal pleiotropy was assessed using the MR-Egger regression intercept [22].

Since BMI and WHR are correlated covariates to each other, collider bias is likely to affect our results. To avoid this problem, we performed the multivariable MR method [23] to adjust BMI for WHR, and likewise, to adjust WHR for BMI. In addition, we included Helicobacter Pylori (H.P.) infection, type 2 diabetes (T2D), dyslipidemia, smoking, and alcohol use in multivariable MR to determine whether the causal association between adiposity and PUD, if any, was mediated by these traditional risk factors. Summary statistic for H.P. infection was derived from UK Biobank database (http://www.nealelab.is/uk-biobank, ID: ukb-b-531), T2D from the GWAS conducted by DIAGRAM consortium (452,244 individuals; 81,412 cases) [24], circulating lipid levels (LDL-C, HDL-C, TC, and TG) from the Global Lipids Genetics Consortium (188,578 individuals) [25], and smoking and alcohol use from the GWAS & Sequencing Consortium of Alcohol and Nicotine use (1,232,091 individuals) [26].

We calculated the statistical power using a publicly available tool (https://shiny.cnsgenomics.com/mRnd/) based on a type 1 error of 5% [27]. There was over 80% power to detect an OR of 1.09 for the BMI-PUD association, and 1.12 for the WHR-PUD association in the primary analyses; an OR of 1.15 for the BMI-PUD association, and 1.25 for the WHR-PUD association in the replication analyses. The estimates were considered significant at *P* < 0.025 (0.05/2 exposures). MR analyses were performed using packages including TwoSampleMR [28], MendelianRandomization [29], and MR-PRESSO [21] within software R (version 4.1.0).

## Results

All IVs in the present study had F-statistics above the threshold of 10, suggesting sufficient IV strength for MR analyses. These SNPs were estimated to account for 5.51% and 3.43% of the phenotypic variation of BMI and WHR, respectively (Additional file 1: Table S2 and S3). In the replication analyses, genetic variants IVs explained 2.32% and 0.78% of the variation of BMI and WHR, respectively (Additional file 1: Table S4 and S5). The selected SNPs for PUD were estimated to account for 2.32% of the variation of PUD (Additional file 1: Table S7).

**Fig. 1:**
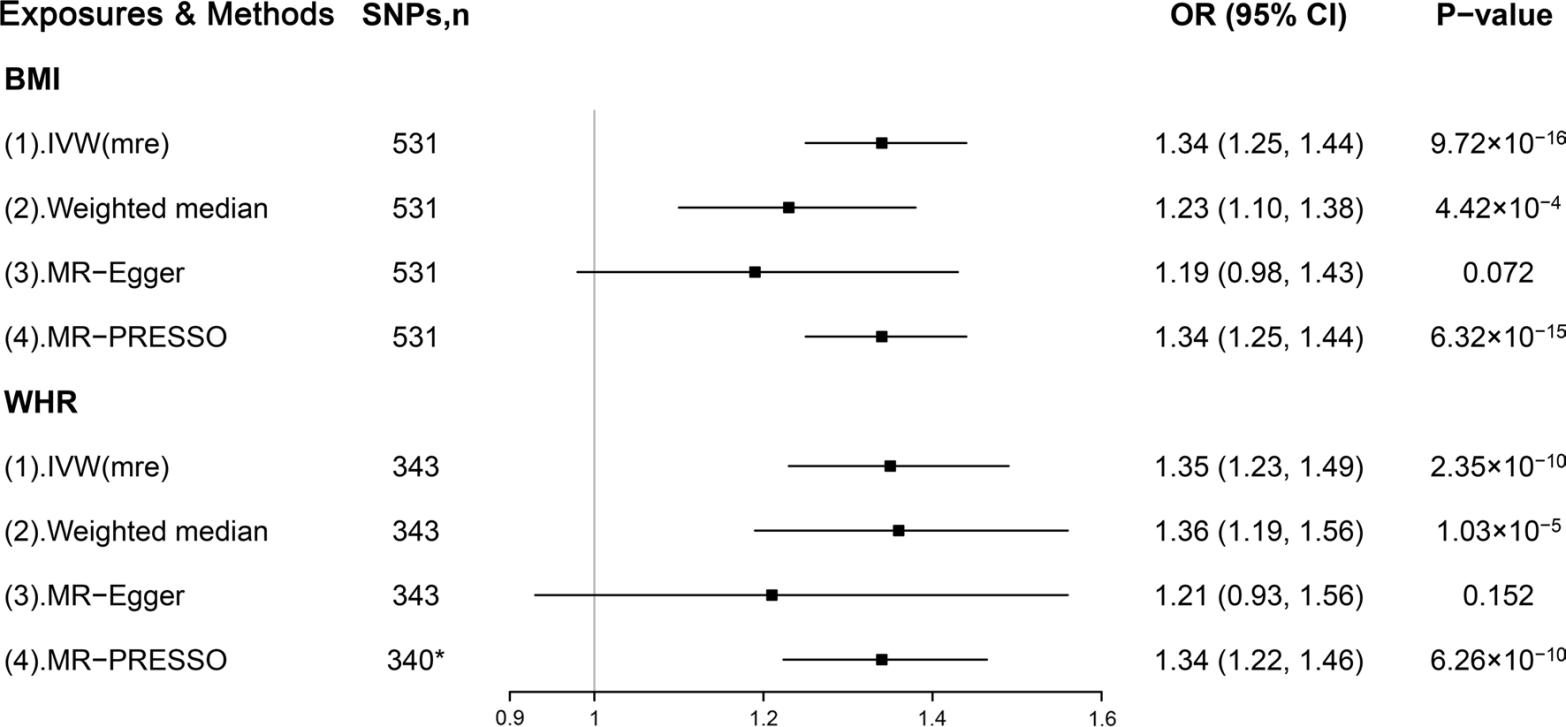
Associations of genetically determined BMI and WHR with PUD risk. BMI: body mass index; WHR: waist-to-hip ratio; PUD, peptic ulcer disease; SNP, single nucleotide polymorphism; OR, odds ratio; CI, confidence interval; IVW (mre), multiplicative random-effects inverse-variance weighted; MR-Egger, Mendelian Randomization-Egger; MR-PRESSO, MR-Pleiotropy Residual Sum and Outlier. Datasets for BMI and WHR were extracted from GWAS conducted by Pulit et al. *Excluding the outliers for WHR (rs1680490, rs668871, and rs6861681)

For per SD increase in genetically determined BMI (~ 4.8 kg/m^2^) there was 34% increased odds of PUD (95% confidence interval [CI], 25–44%; *P* = 9.72 × 10^–16^; Fig. 1 and Additional file 1: Fig S1). In an analogous analysis, we observed that each 0.09 ratio higher WHR was associated with a 35% increase in risk for PUD (95% CI, 23–49%; *P* = 2.35 × 10^–10^; Fig. 1 and Additional file 1: Fig S1). The results remained broadly consistent in the complementary analyses such as weighted median and MR-Egger regression (Fig. 1). There were 3 outliers for WHR (rs1680490, rs668871, and rs6861681) identified by the MR-PRESSO method. The results did not substantially change after correcting for these outliers (Fig. 1). Importantly, little evidence for heterogeneity and horizontal pleiotropy was found (Additional file 1: Table S8).

Multivariable MR demonstrated that the effect of BMI on PUD was attenuated upon adjustment for WHR (Table 2). Nevertheless, the causal association of WHR with PUD persisted after adjusting for BMI (Table 2). Besides, both BMI-PUD and WHR-PUD associations remained consistent following adjustment for genetically determined H.P. infection, T2D, circulating lipid levels, smoking, or alcohol use (Table 2). Furthermore, the results were robust under adjustment for all these lifestyle factors (Table 2).

**Table 2:** Multivariable Mendelian randomization for the associations between obesity traits and PUD adjusting for potential mediators

Model	BMI		WHR	
	OR (95% CI)	*P*-value	OR (95% CI)	*P*-value
Unadjusted model	1.34 (1.25, 1.44)	9.72 × 10^–16^	1.35 (1.23, 1.49)	2.35 × 10^–10^
Adjusted for WHR	1.19 (0.99, 1.43)	0.070	–	–
Adjusted for BMI	–	–	1.23 (1.06, 1.41)	0.005
Adjusted for H.P. infection	1.30 (1.18, 1.43)	3.17 × 10^–8^	1.31 (1.16, 1.48)	8.89 × 10^–6^
Adjusted for T2D	1.34 (1.23, 1.46)	3.03 × 10^–12^	1.39 (1.24, 1.55)	1.56 × 10^–8^
Adjusted for LDL-C	1.33 (1.23, 1.44)	8.68 × 10^–13^	1.31 (1.18, 1.46)	9.15 × 10^–7^
Adjusted for HDL-C	1.35 (1.23, 1.49)	3.14 × 10^–10^	1.40 (1.25, 1.57)	1.23 × 10^–8^
Adjusted for TC	1.33 (1.23, 1.44)	1.53 × 10^–13^	1.32 (1.19, 1.46)	1.91 × 10^–7^
Adjusted for TG	1.34 (1.22, 1.46)	1.16 × 10^–10^	1.38 (1.22, 1.55)	1.56 × 10^–7^
Adjusted for smoking	1.25 (1.15, 1.35)	2.86 × 10^–8^	1.27 (1.15, 1.40)	3.70 × 10^–6^
Adjusted for alcohol use	1.34 (1.25, 1.44)	2.52 × 10^–16^	1.36 (1.24, 1.49)	1.83 × 10^–10^
Adjusted for all lifestyle factors*	1.16 (1.03,1.31)	0.013	1.20 (1.03,1.39)	0.022

*BMI* Body Mass Index; *WHR* Waist-to-hip ratio; *H.P.* Helicobacter Pylori; *T2D* type 2 diabetes mellitus; *LDL-C* low-density lipoprotein cholesterol; *HDL-C* high-density lipoprotein cholesterol; *TC* total cholesterol; *TG* triglyceride

*Factors including H.P infection, T2D, LDL-C, smoking, and alcohol use. Restricted to LDL-C to avoid collinearity with HDL-C, TC and TG levels

**Fig. 2:**
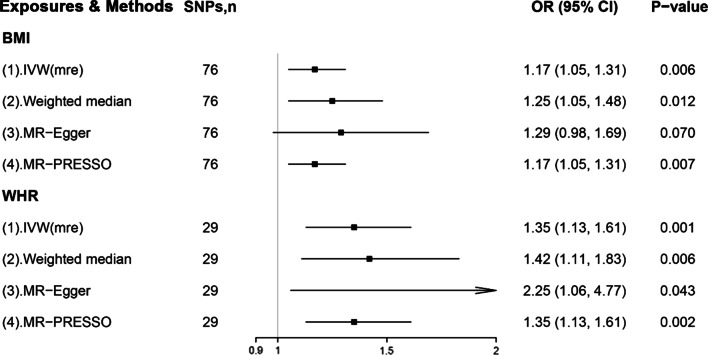
Replication analyses showing the associations of genetically determined BMI and WHR with PUD risk. BMI: body mass index; WHR: waist-to-hip ratio; PUD, peptic ulcer disease; SNP, single nucleotide polymorphism; OR, odds ratio; CI, confidence interval; IVW (mre), multiplicative random-effects inverse-variance weighted; MR-Egger, Mendelian Randomization-Egger; MR-PRESSO, MR-Pleiotropy Residual Sum and Outlier. Datasets for BMI and WHR were extracted from GWAS conducted by Locke et al. and Shungin et al., respectively

In the replication analyses, we used IVs based on summary-level data for both obesity traits from individuals included in the GIANT consortium [15, 16]. IVW analysis showed that genetically-predicted higher BMI (per SD) was associated with an 17% increase in risk for PUD (95% CI = 5–31; *P* = 0.006; Fig. 2 and Additional file 2: Fig S2). The MR estimate for WHR-PUD association was also positive and, notably, of a greater magnitude; Genetically-predicted higher WHR was associated with 35% higher risk of PUD (95% CI = 13–61%; *P* = 0.001; Fig. 2 and Additional file 2: Fig S2). Complementary analyses returned broadly consistent results (Fig. 2 and Additional file 2: Fig S2). No pleiotropic outliers were detected by the MR-PRESSO test. The risk of heterogeneity and horizontal pleiotropy should be low according to multiple tests listed in Additional file 1: Table S9.

**Fig. 3:**
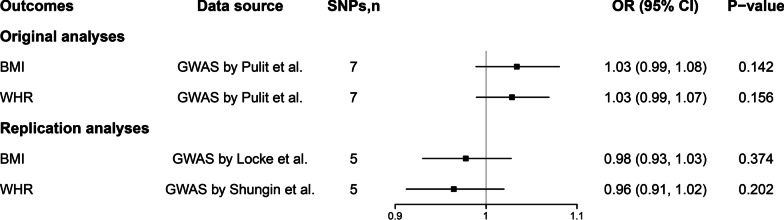
Associations of genetically determined PUD with risk of BMI and WHR. BMI: body mass index; WHR: waist-to-hip ratio; PUD, peptic ulcer disease; SNP, single nucleotide polymorphism; OR, odds ratio; CI, confidence interval

Reverse MR analyses provided no evidence for the causal effect of PUD on BMI or WHR (Fig. 3 and Additional file 2: Fig S3). The association pattern persisted when different datasets for BMI and WHR were used (Fig. 3 and Additional file 2: Fig S4). Besides, complementary analyses yielded broadly consistent results (Additional file 1: Table S10). SNP heterogeneity was high for BMI and WHR in the original analyses but not in the replication analyses. (Additional file 1: Table S11 and S12). MR Egger regression indicated a low risk of pleiotropy in both the original and replication analyses (Additional file 1: Table S11 and S12).

## Discussion

This bidirectional MR study demonstrated that genetically predicted obesity (notably abdominal obesity) was causally associated with PUD risk. The results were consistent across complementary analyses and survived adjustment for several traditional risk factors. Besides, no evidence was found to support the causal effect of PUD on obesity.

The association between obesity and PUD remain a subject of ongoing debate. Generally speaking, PUD is divided into gastric ulcers (GU) and duodenal ulcers (DU). Our results collaborated with a series of previous observational studies. Evidence from a cross-sectional study reported a positive association between obesity and GU (but not DU) (OR = 4.15; 95% CI, 1.31–13.13) among northern Sweden individuals [7]. A retrospective cohort study enrolling 32,472 Korean individuals found a pattern of relationships of higher BMI with increased risk of GU (OR, 1.32; 95% CI, 1.16–1.49; *P* < 0.001), but not with the risk of DU [8]. Likewise, another large prospective cohort study with 47,120 U.S. individuals reported an increased prevalence of GU in subjects with higher BMI upon multivariate adjustment (OR, 1.83; 95% CI, 1.20–2.78; *P* < 0.01) [5]. Similarly, the same research group showed that BMI was not associated with a greater tendency in suffering from DU [5]. In this study, we provided evidence supporting the positive association of obesity with PUD risk in European populations and demonstrated a potential causality of this relationship. Considering that significance was observed only in the BMI-GU relationship in previous studies, we speculated that the positive results observed in this MR study may be largely attributable to the fact that BMI increased the risk of GU, but not DU. Future MR study using separate GWAS datasets for GU and DU should shed light on this important issue.

The multivariable MR study suggested a great attenuation of the association between BMI and PUD after adjusting for WHR. Indeed, BMI is considered an imprecise obesity classification method since it ignores the distribution of adiposity and cannot distinguish between lean body mass and fat mass [30, 31]. Therefore, it may be a relatively poor tool for exploring associations between obesity and diseases. On the other hand, our results demonstrated that the association between WHR and PUD was not significantly attenuated after adjusting for BMI. As previously reported, WHR is an independent biological tool for abdominal adiposity and visceral fat measurement [32]. The results from the multivariable MR specifically demonstrated that the causal association between WHR and PUD was independent of BMI. In other words, individuals with higher abdominal adiposity are more likely to suffer from PUD, even if their BMI is relatively low.

The pathophysiology of PUD has been associated with the use of NSAIDs and *Helicobacter Pylori* infection. However, the underlying mechanisms linking obesity to PUD is not yet well-elucidated. Observationally, obesity was strongly associated with PUD in non-NSAIDs/aspirin users and *Helicobacter Pylori*-negative subjects [5]. Therefore, the observed causal effect of obesity on PUD is likely to be independent of anti-inflammatory drug use or *Helicobacter Pylori* infection. Recent studies have linked obesity to mucosal dysfunction [33, 34], which is one of the potential mechanisms associated with PUD [35]. In our study, we performed multivariable MR analyses to explore potential mediators. Obesity has been associated with metabolic diseases like T2D [36] and dyslipidemia [37], and unhealthy behaviors like smoking [38] and alcohol use [39]. However, this MR study demonstrated that the association of obesity with PUD risk persisted upon adjustment for these risk factors. In addition, obesity-induced alteration in gut microbiota [3, 4] and gastrointestinal inflammation [40] are also likely to mediate the causal associations. However, detailed research is insufficient. Further studies into how obesity is involved in the development of PUD are warranted.

There are three issues that can violate the MR assumption and lead to biased causal inferences: (1) biological mechanism; (2) genetic coinheritance; and (3) population effects. Pleiotropy represents one of the most important biological mechanisms. In the present study, MR-Egger intercept test was carried out to assess pleiotropy. As shown in Additional file 1: Table S8, S9, S11 and S12, there was no evidence for pleiotropy for all associations considered. Secondly, we ruled out the effect of non-Mendelian inheritance by performing the clumping process with R^2^ threshold of < 0.001 and window size of 10,000 kb. Thereby, the influence of genetic coinheritance would be minimal in this study. Third, the population stratification is unlikely to bias our results since all of the participants involved in these original GWASs were of European ancestry.

The MR design that we used is the first strength of this study, which can minimize biases such as residual confounders and reverse causation. Reverse causation describes a scenario where post-event measurement of the exposure can be affected by the outcome event. For example, one can hardly tell whether event A cause higher risk of event B or event B increased incidence of event A in the observational studies. The MR approach can avoid reverse causation because genetic variants were randomly assorted during conception and unlikely to be influenced by disease status. Besides, we here performed reverse MR analyses from PUD to obesity and no evidence was found for the causal association in this direction, which further minimized the influence of reverse causation. Second, the present study leveraged a high statistical power (above the threshold of 80%) enabled by the use of the largest GWAS meta-analyses to date. Third, all the complementary approaches returned consistent results, strengthening the causal inference. Finally, the analyses were less likely to be affected by the bias of population structure since they were restricted to individuals of European descent.

However, several limitations deserved consideration. First, we did not evaluate the associations between obesity and different subtypes of PUD (GU and DU) due to a lack of related GWASs. Second, the potential non-linear association cannot be assessed since this study relies on summary statistics. Third, sample overlap in the GWASs of obesity and PUD (UK Biobank mainly) are likely to bias the causal estimates and inflate Type 1 error rates [41] in the primary analysis. However, given that all genetic variants that we used were confirmed to be strong (F statistics > 10), we did not expect substantial bias here [41]. In addition, the results were consistent in the replication analyses where no sample overlap was present between the exposure and outcome. Finally, the limitation of participants in this study to Europeans might limit the generalizability of this study.

## Conclusions

In the present study, we provided genetic evidence showing that obesity (notably abdominal obesity) is causally associated with increased PUD risk. Programs aimed at weight loss may play an important role in preventing PUD.

## Supplementary Information

**Additional file 1. Tabel S1**. Proxies for SNPs not found in the outcome dataset (Forward MR); **Tabel S2**. Characteristics of the genetic variants associated with body mass index; **Tabel S3**. Characteristics of the genetic variants associated with waist-to-hip ratio; **Tabel S4**. Characteristics of the genetic variants associated with body mass index in replication analyses; **Tabel S5**. Characteristics of the genetic variants associated with waist-to-hip ratio in replication analyses; **Tabel S6**. Proxies for SNPs not found in the outcome dataset (Reverse MR); **Tabel S7**. Characteristics of the genetic variants associated with peptic ulcer disease; **Tabel S8**. Evaluation of heterogeneity and directional pleiotropy using different methods (Forward MR); **Tabel S9**. Evaluation of heterogeneity and directional pleiotropy in replication analyses (Forward MR); **Table S10**. Associations of genetic predisposition to PUD with risk of obesity in complementary Mendelian Randomization analyses (Reverse MR); **Tabel S11**. Evaluation of heterogeneity and directional pleiotropy using different methods (Reverse MR); **Tabel S12**. Evaluation of heterogeneity and directional pleiotropy in replication analyses (Reverse MR).

**Additional file 2. Figure S1**. Scatter plots for MR estimates of BMI (A) and WHR (B) on PUD; **Figure S2**. Scatter plots for MR estimates of BMI (A) and WHR (B) on PUD in the replication analysis; **Figure S3**. Scatter plots for MR estimates of PUD on BMI (A) and WHR (B); **Figure S4**. Scatter plots for MR estimates of PUD on BMI (A) and WHR (B) in the replication analysis.

## Data Availability

The summary statistics of GWAS for peptic ulcer disease are derived from a GWAS conducted by Yeda Wu et al. (https://cnsgenomics.com/content/data); Full GWAS summary statistics for BMI and WHR are publicly available through https://doi.org/10.5281/zenodo.1251813; In replication analysis, summary level data for BMI and WHR can be accessed at https://portals.broadinstitute.org/collaboration/giant/index.php/GIANT_consortium_data_files; Summary statistics for H.P. infection was derived from UK Biobank database (http://www.nealelab.is/uk-biobank, ID: ukb-b-531); Summary statistics for type 2 diabetes is available at the DIAGRAM consortium website (http://diagram-consortium.org/); Summary statistics for LDL-C, HDL-C, TC and TG are available through http:/www.sph.umich.edu/csg/abecasis/lipids2013/; Summary statistics for smoking and alcohol use can be download from https://conservancy.umn.edu/handle/11299/201564.

## Abbreviations

PUD: Peptic ulcer disease
MR: Mendelian randomization
BMI: Body mass index
WHR: Waist-to-hip ratio
GWAS: Genome-wide association study
OR: Odds ratio
CI: Confidence interval
NSAIDs: Non-steroidal anti-inflammatory drugs
IVs: Instrumental instruments
SNPs: Single nucleotide polymorphisms
MR-PRESSO: MR-pleiotropy residual sum and outlier
T2D: Type 2 diabetes
LDL-C: Low-density lipoprotein cholesterol
HDL-C: High-density lipoprotein cholesterol
TC: Total cholesterol
TG: Triglycerides
GU: Gastric ulcer
DU: Duodenal ulcer
