# Supplementary material for: Genetic liability to obesity and peptic ulcer disease: a Mendelian randomization study
Source: BMC Med Genomics. 2022 Oct 4;15:209. doi: 10.1186/s12920-022-01366-x (PMC9533532; doi:10.1186/s12920-022-01366-x)
Supplement: Supplementary file 2 — Additional file 2. Figure S1. Scatter plots for MR estimates of BMI (A) and WHR (B) on PUD; Figure S2. Scatter plots for MR estimates of BMI (A) and WHR (B) on PUD in the replication analysis; Figure S3. Scatter plots for MR estimates of PUD on BMI (A) and WHR (B); Figure S4. Scatter plots for MR estimates of PUD on BMI (A) and WHR (B) in the replication analysis. [file 12920_2022_1366_MOESM2_ESM.docx]

**Figure S1.**


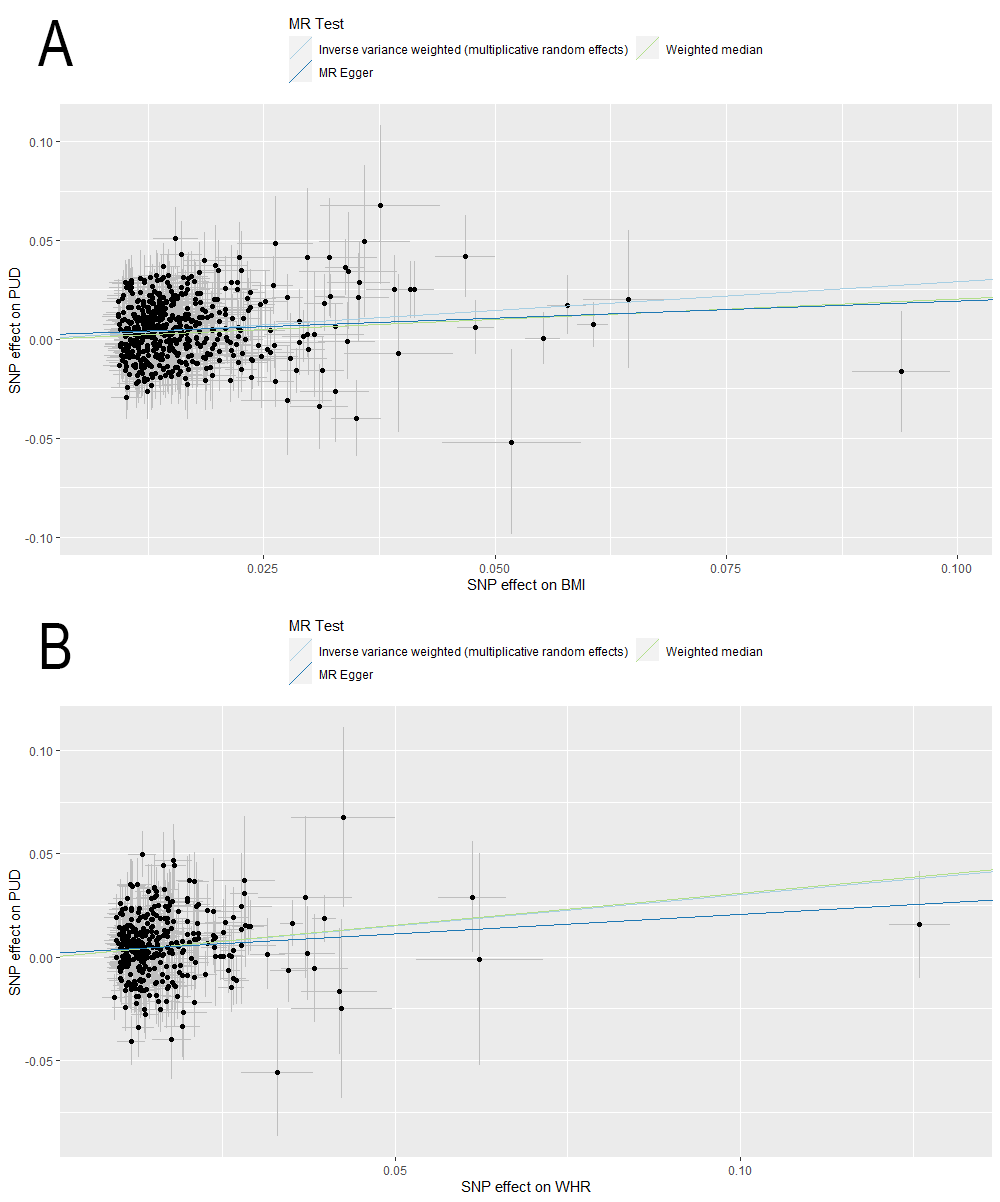


**Figure S1.** Scatter plots for MR estimates of BMI (A) and WHR (B) on PUD.

BMI: body mass index; WHR: waist-to-hip ratio; PUD, peptic ulcer disease; MR-Egger, Mendelian Randomization-Egger.

Datasets for BMI and WHR were extracted from GWAS conducted by pulit et al.

**Figure S2.**


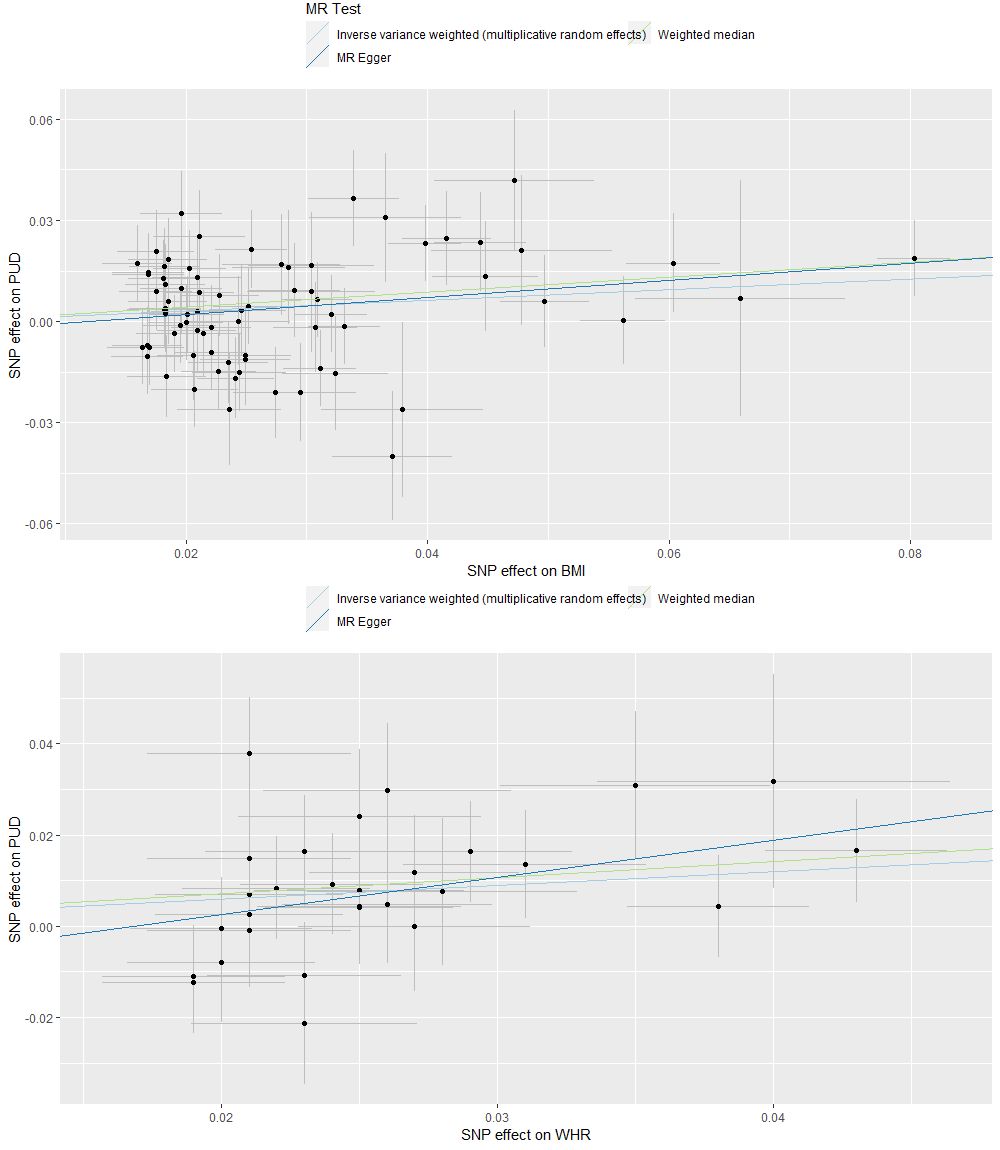


**Figure S2.** Scatter plots for MR estimates of BMI (A) and WHR (B) on PUD in the replication analysis.

BMI: body mass index; WHR: waist-to-hip ratio; PUD, peptic ulcer disease; MR-Egger, Mendelian Randomization-Egger.

Datasets for BMI and WHR were extracted from GWAS conducted by Locke et al. and Shungin et al., respectively.

**Figure S3.**


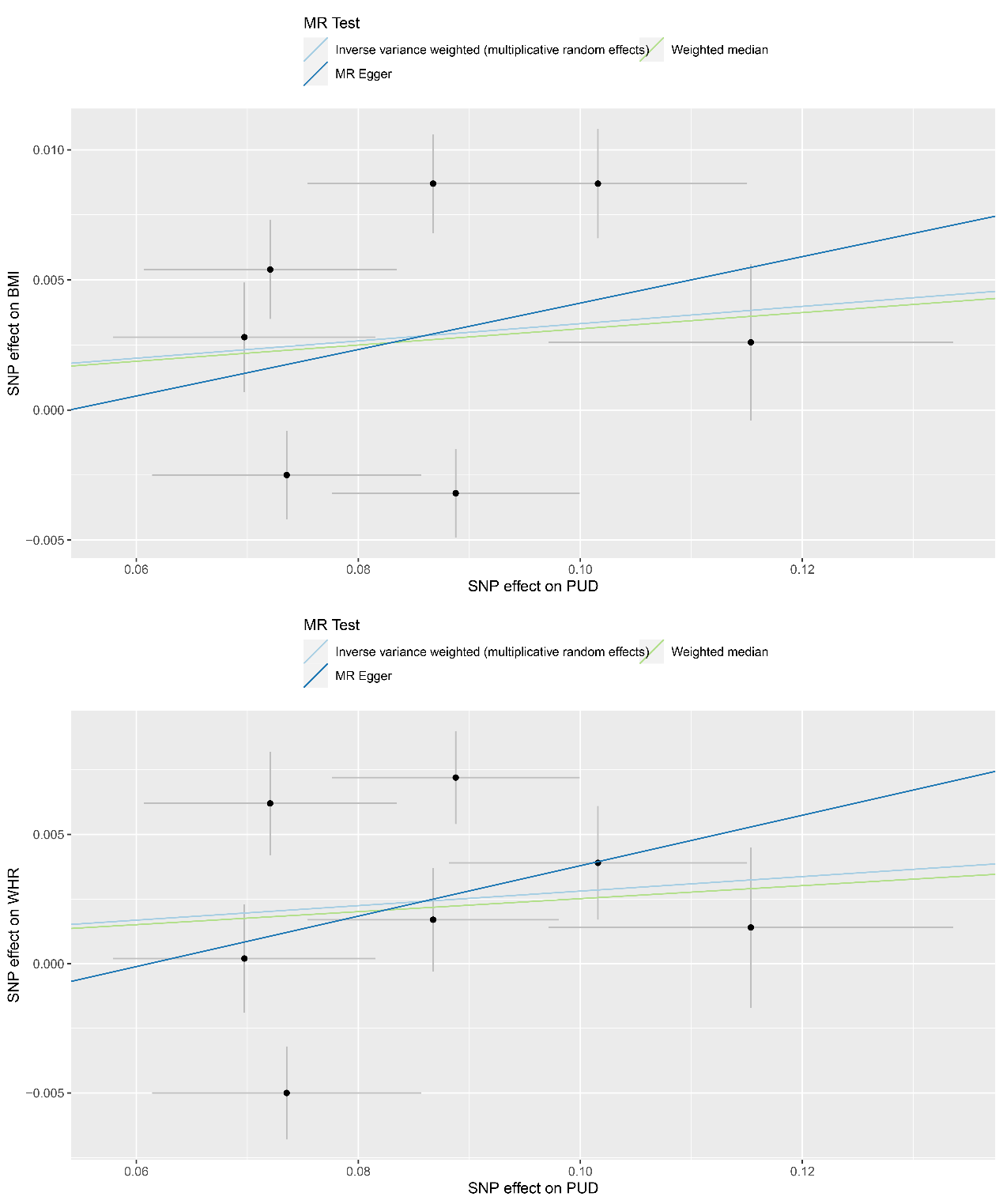


**Figure S3.** Scatter plots for MR estimates of PUD on BMI (A) and WHR (B).

BMI: body mass index; WHR: waist-to-hip ratio; PUD, peptic ulcer disease; MR-Egger, Mendelian Randomization-Egger.

Datasets for BMI and WHR were extracted from GWAS conducted by pulit et al.

**Figure S4.**


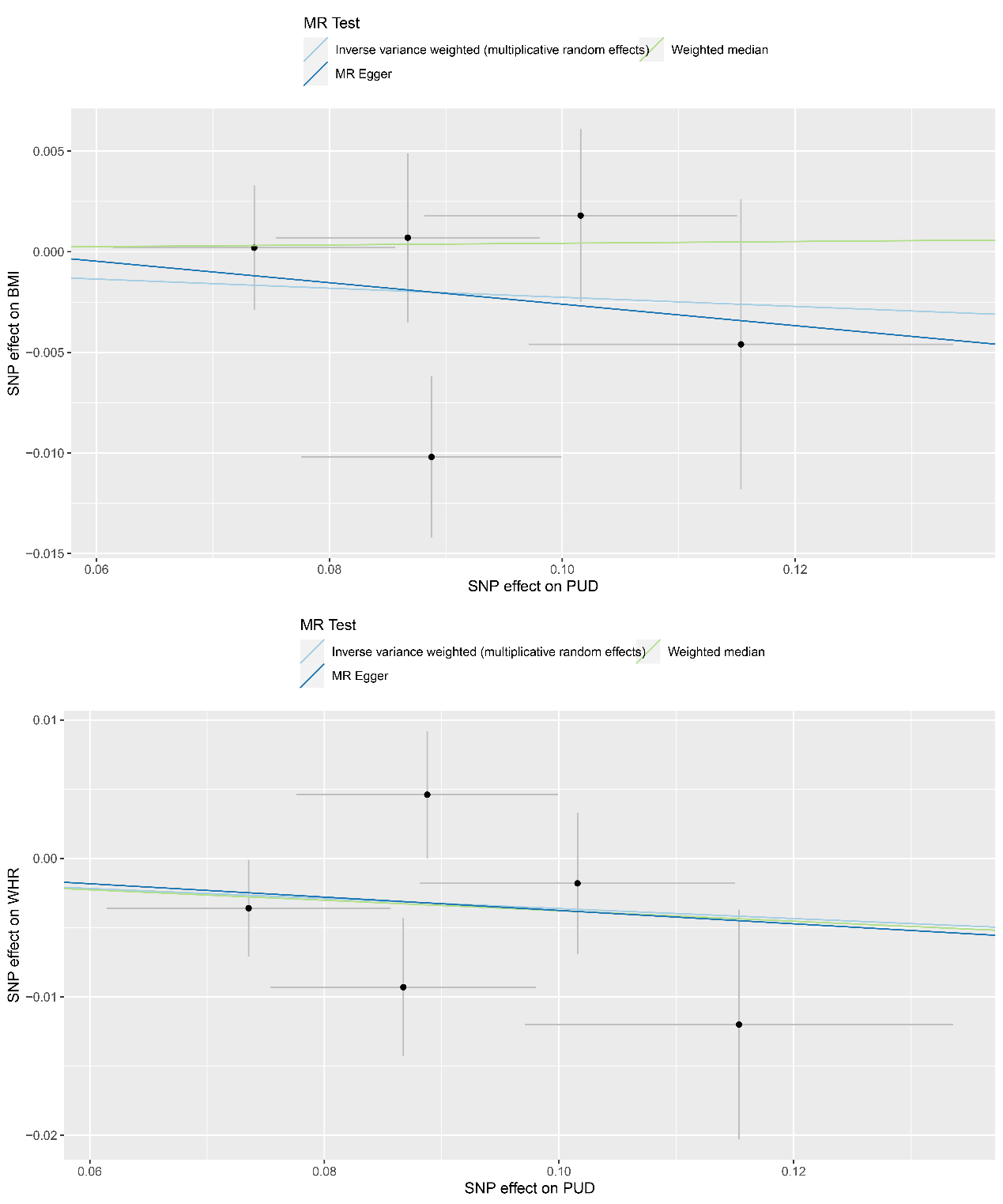


**Figure S4.** Scatter plots for MR estimates of PUD on BMI (A) and WHR (B) in the replication analysis.

BMI: body mass index; WHR: waist-to-hip ratio; PUD, peptic ulcer disease; MR-Egger, Mendelian Randomization-Egger.

Datasets for BMI and WHR were extracted from GWAS conducted by Locke et al. and Shungin et al., respectively.
